# Using Machine Learning Techniques to Predict Factors Contributing to the Incidence of Metabolic Syndrome in Tehran: Cohort Study

**DOI:** 10.2196/27304

**Published:** 2021-09-02

**Authors:** Firoozeh Hosseini-Esfahani, Behnaz Alafchi, Zahra Cheraghi, Amin Doosti-Irani, Parvin Mirmiran, Davood Khalili, Fereidoun Azizi

**Affiliations:** 1 Department of Clinical Nutrition and Dietetics Faculty of Nutrition Sciences and Food Technology, National Nutrition and Food Technology Research Institute Shahid Beheshti University of Medical Sciences Tehran Iran; 2 Department of Biostatistics School of Public Health Hamadan University of Medical Sciences Hamadan Iran; 3 Modeling of Noncommunicable Diseases Research Center Hamadan University of Medical Sciences Hamadan Iran; 4 Department of Epidemiology School of Public Health Hamadan University of Medical Sciences Hamadan Iran; 5 Health and Research Center for Health Sciences Hamadan University of Medical Sciences Hamadan Iran; 6 Prevention of Metabolic Disorders Research Center Research Institute for Endocrine Sciences Shahid Beheshti University of Medical Sciences Tehran Iran; 7 Department of Biostatistics and Epidemiology Research Institute for Endocrine Sciences Shahid Beheshti University of Medical Sciences Tehran Iran

**Keywords:** metabolic syndrome, Tehran Lipid and Glucose Study, data mining

## Abstract

**Background:**

Metabolic syndrome (MetS), a major contributor to cardiovascular disease and diabetes, is considered to be among the most common public health problems worldwide.

**Objective:**

We aimed to identify and rank the most important nutritional and nonnutritional factors contributing to the development of MetS using a data-mining method.

**Methods:**

This prospective study was performed on 3048 adults (aged ≥20 years) who participated in the fifth follow-up examination of the Tehran Lipid and Glucose Study, who were followed for 3 years. MetS was defined according to the modified definition of the National Cholesterol Education Program/Adult Treatment Panel III. The importance of variables was obtained by the training set using the random forest model for determining factors with the greatest contribution to developing MetS.

**Results:**

Among the 3048 participants, 701 (22.9%) developed MetS during the study period. The mean age of the participants was 44.3 years (SD 11.8). The total incidence rate of MetS was 229.9 (95% CI 278.6-322.9) per 1000 person-years and the mean follow-up time was 40.5 months (SD 7.3). The incidence of MetS was significantly (*P*<.001) higher in men than in women (27% vs 20%). Those affected by MetS were older, married, had diabetes, with lower levels of education, and had a higher BMI (*P*<.001). The percentage of hospitalized patients was higher among those with MetS than among healthy people, although this difference was only statistically significant in women (*P*=.02). Based on the variable importance and multiple logistic regression analyses, the most important determinants of MetS were identified as history of diabetes (odds ratio [OR] 6.3, 95% CI 3.9-10.2, *P*<.001), BMI (OR 1.2, 95% CI 1.0-1.2, *P*<.001), age (OR 1.0, 95% CI 1.0-1.03, *P*<.001), female gender (OR 0.5, 95% CI 0.38-0.63, *P*<.001), and dietary monounsaturated fatty acid (OR 0.97, 95% CI 0.94-0.99, *P*=.04).

**Conclusions:**

Based on our findings, the incidence rate of MetS was significantly higher in men than in women in Tehran. The most important determinants of MetS were history of diabetes, high BMI, older age, male gender, and low dietary monounsaturated fatty acid intake.

## Introduction

Metabolic syndrome (MetS), a major contributor to cardiovascular disease and diabetes, is considered to be among the most common public health problems worldwide [1]. According to the World Health Organization and the International Diabetes Federation, MetS is defined as the simultaneous occurrence of three of the following five medical conditions: abdominal obesity, high blood pressure, hyperglycemia, high triglyceride levels, and low high-density lipoprotein cholesterol (HDL-C) levels [2].

The incidence of MetS is estimated to be 34% in the United States [3], 12%-37% in Asian countries [4], and 12%–26% in European populations [5]. In Iran, the overall pooled prevalence and incidence rate of MetS among the general population was reported to be 0.26 (95% CI 0.25-0.29) and 97.96 per 1000 person-years (95% CI 75.98-131.48), respectively, and was higher in women living in urban areas and in men living in rural areas.

The overall pooled prevalence of MetS was higher in urban areas compared to rural areas (0.39 vs 0.26) and the pooled prevalence of MetS was higher in women than in men (0.34 vs 0.22) [6].

According to previous studies, the etiology of MetS is controlled by several risk factors, including abdominal obesity, insulin resistance, glucose tolerance disorder, hypertension, genetic factors, psychosocial stressors, and nutritional and diet factors [7-11]. Previous studies have often investigated the predictive factors using classical approaches and neglected the interpretability of the results. For example, among the explanatory variables, the risk/protective factors have a more important role in the outcomes. One of the simplest and very common ranking techniques is random forest (RF), which is a data-mining approach. The most important features of this model are simplicity and interpretation of the model, flexibility in applying a large number of predictor variables, working with an infinite sample size, and determination of important variables in predicting the outcome. The RF model is also useful when predictor variables are nonlinear concerning disease, because there is no assumption or any constraint on the form of the relationships [12-14]. Considering the high prevalence of MetS and its importance in cardiovascular disease, identifying and ranking the most important nutritional and nonnutritional factors in the occurrence of MetS is an essential analysis with respect to public health. Data-mining methods are strong tools in predicting different outcomes and emphasizing interpretability with benefits for precision prediction. Hence, we aimed to identify and rank the most important nutritional and nonnutritional factors in the occurrence of MetS among the general population of Tehran, Iran, using the RF data-mining method.

## Methods

### Design and Participants

This prospective study (Code: IR.UMSHA.REC.1398.864) was performed under the framework of the Tehran Lipid and Glucose Study, a population-based study to determine risk factors for noncommunicable diseases in a sample of residents of District 13 of the Tehran metropolis [15,16]. The first examination survey was performed from 1999 to 2001 on 15,005 individuals aged ≥3 years. Subsequently, follow-up examinations were performed every 3 years (2002-2005, 2005-2008, 2008-2011, 2011-2014, and 2015-2018) to identify recently developed diseases (see Multimedia Appendix 1 for more details on the survey).

**Figure 1:**
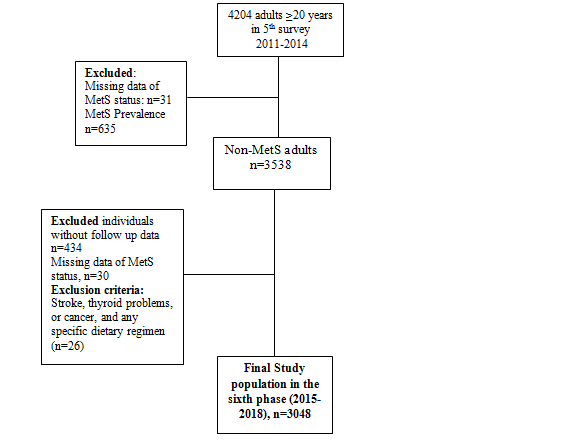
Flowchart of the study participants (MetS: metabolic syndrome; TLGS: Tehran Lipid and Glucose Study).

In the fifth follow-up examination (2011-2014), 4204 adults (aged ≥20 years) participated. These participants completed the Food Frequency Questionnaire (FFQ), and their dietary data were available. The exclusion criteria in this study were as follows: individuals diagnosed with MetS (n=635); people with missing data regarding MetS status (n=61); no follow-up (n=434); stroke, thyroid, or cancer complications (n=18); and following a specific dietary regimen (n=8). Finally, 3048 adults without MetS at baseline were included in the study (Figure 1). All invited participants signed the informed written consent form. The study was performed in adherence with the Declaration of Helsinki. The ethics committee of the Research Institute for Endocrine Sciences, Shahid Beheshti University of Medical Sciences approved the study protocol.

### Outcomes

MetS was defined according to the modified definition of the National Cholesterol Education Program/Adult Treatment Panel III [17,18] as having at least three of the following symptoms simultaneously: (1) abdominal obesity (waist circumference >90 cm in both genders); (2) serum HDL-C level <40 mg/dl in men and <50 mg/dl in women or taking HDL-C–elevating drugs; (3) hypertension (systolic blood pressure ≥130 mmHg, diastolic blood pressure ≥85 mmHg, or taking antihypertensive drugs); (4) hyperglycemia (fasting blood glucose ≥100 mg/dl or taking hypoglycemic drugs); and (5) hypertriglyceridemia (serum triglyceride level ≥150 mg/dl or taking triglyceride-lowering drugs).

### Risk Factor Assessment

In this study, the FFQ was used to measure the exact amount of food intake. The FFQ is a valid and reliable tool for measuring 147 food items (Multimedia Appendix 2) [18]. Trained nutritionists helped the participants to complete the questionnaires through face-to-face interviews. The usual average size of each food item was explained to each participant, considering the frequency of consumption on a daily, weekly, or monthly basis [18,19]. Portion sizes were converted to grams using household measures. Due to the incompleteness of the Iranian food composition table, the United States Department of Agriculture food consumption table was used to analyze foods in terms of their macro- and micronutrients [20,21]. A literature review was performed to select effective nutrients for MetS [22-24].

Weight was measured to the nearest 100 g using digital scales (Seca, Hamburg, Germany) while subjects were minimally clothed and not wearing shoes. Height was measured to the nearest 0.5 centimeter using a stadiometer while the subjects were in a standing position, with their shoulders in normal alignment and without shoes. Information on age, gender, marital status (single, divorced, widowed), history of hospitalization in the previous 3 months, history of cancer, education (primary, intermediate, high school, and academic education), and smoking (never smoked, past smoker, current smoker) was collected using a general information questionnaire.

### Statistical Analysis

The *χ^2^* test and *t* test were applied to explore the differences in qualitative and quantitative variables between groups. Since the data-mining approach cannot reveal the direction of the association of variables on the outcome, multiple logistic regression was used to estimate the adjusted effect of variables. The backward-selection method was applied to choose the variables in this model. To remove variables from the model, the *P* value threshold was set to .20. R software (version 3.6.1) with the *randomForest* and *caret* packages was used for data analysis.

### RF Analysis

RF, proposed by Leo Breiman [25], is an ensemble learning method that grows many classification trees. A random sample with replacement of the original training dataset was used to construct the trees in RF. The algorithm only searches across a random subset of the input variables at each node to determine the best split. Finally, RF chooses the class with the most votes over all the trees in the forest [25]. RF has exhibited superior performance over other machine-learning methods such as support vector machine, artificial neural network, and k-nearest neighbor [26-28].

Moreover, although most machine-learning classifiers are useful for classifying, they do not provide any insight into the most important variables based on the derived classifier. However, RF provides variable importance measurements that can be used in model interpretation [26]. The most common method to find the most important variable is to use the mean decrease in accuracy and the mean decrease in the Gini index [26,29].

### Evaluation Criteria

Our dataset consisted of 2259 adults (after removing variables with missing data) divided into training and testing sets. We randomly chose 70% of the data as the training set and the remaining 30% as the test set. The RF classifier was trained using the training dataset. The test dataset was used to evaluate the performance of the method. To evaluate the performance of the RF classifier, we used several evaluation criteria of sensitivity, specificity, negative predictive value (NPV), positive predictive value (PPV), negative likelihood ratio (LR–), and positive likelihood ratio (LR+) (see Multimedia Appendix 3).

## Results

### Baseline Characteristics

The dataset included 3048 adults, 701 (22.9%) of whom developed MetS and 2347 (77.1%) of whom did not develop MetS. The mean age of the participants at baseline was 44.3 years (SD 11.8). The total MetS incidence rate was 229.98 (95% CI 278.6-322.9) per 1000 person-years. The incidence of MetS was significantly higher in men than in women (27% vs 20%). In both genders, those affected by MetS were older, married, had diabetes, and a lower level of education (*P*<.001) than their counterparts. In men, a greater frequency of smokers were affected by MetS (*P*=.05), and the percentage of hospitalized subjects in patients with MetS syndrome was higher than that among healthy people, although this difference was only statistically significant in women (*P*=.02) (Table 1).

The distribution of the characteristics of subjects in the training and test datasets is presented in Table 2. The results showed no statistically significant differences between the training and test sets.

**Table 1:** Baseline characteristics of participants who developed and did not develop metabolic syndrome (MetS) by gender.

Variables		Men				Women				All		
		No MetS (n=838)	MetS (n=311)	*P* value^a^	No MetS (n=1509)		MetS (n=390)	*P* value	No MetS (n=2347)		MetS (n=701)	*P* value
Age (years), mean (SD)		45.8 (13.6)	47.1 (12.9)	.08	41.9 (10.1)		51.4 (10.6)	<.001	43.6 (12.1)		49.5 (12.3)	<.001
BMI (kg/m^2^), mean (SD)		25.7 (3.9)	28.3 (3.8)	<.001	26.5 (3.1)		30.4 (4.3)	<.001	26.2 (4.2)		29.5) (4.3)	<.001
**Marital status, n (%)**				.008				.84				.002
	Married	673 (80.4)	271 (87.1)		1201 (79.7)		326 (83.6)		1874 (80.0)		597 (85.2)	
	Single/divorced/widowed	164 (19.6)	40 (12.9)		306 (20.3)		64 (16.4)		470 (20.0)		104 (14.5)	
**Smoking, n (%)**				.05				.18				.66
	Never	662 (79.0)	243 (78.4)		1441 (95.7)		381 (97.7)		2103 (89.7)		624 (89.4)	
	Current/past	176 (21.0)	67 (21.6)		65 (4.3)		9 (2.3)		241 (10.3)		76(10.7)	
**Education level, n (%)**				.003				<.001				<.001
	Higher than diploma	406 (48.6)	121 (39.0)		710 (47.2)		74 (19.4)		1111 (47.7)		195 (28.3)	
	Diploma/below diploma	372 (44.6)	173 (55.8)		717 (47.5)		792 (65.8)		1082 (46.4)		423 (61.3)	
	Illiterate/primary School	57 (6.8)	16 (5.2)		80 (5.3)		56 (14.8)		137 (5.9)		72 (10.4)	
Cancer history, n (%)		3 (0.4)	1 (0.3)	.93	7 (0.5)		4 (1.0)	.19	10 (0.4)		5 (0.7)	.34
Hospitalization, n (%)		15 (1.8)	5 (1.6)	.84	20 (1.3)		12 (3.1)	.02	35 (1.5)		17 (2.4)	.09
Diabetes, n (%)		21 (2.7)	26 (9.1)	<.001	20 (1.5)		66 (18.7)	<.001	41 (1.9)		92 (14.4)	<.001
Systolic blood pressure (mmHg), mean (SD)		112.9 (12.6)	120.69 (14.1)	<.001	104.34 (12.3)		117.84 (15.7)	<.001	107.5 (13.2)		119.1 (15.5)	<.001
Waist circumference (cm), mean (SD)		91.3 (10.5)	98.1 (96.6)	<.001	87.6 (10.4)		98.2 (9.8)	<.001	88.9 (10.6)		98.2 (9.8)	<.001
High triglyceride, n (%)		141 (16.8)	246 (80.0)	<.001	168 (11.1)		299 (76.7)	<.001	309 (13.2)		545 (75.8)	<.001
Physical activity (km/week), mean (SD)		2.8 (0.4)	2.5 (0.4)	.10	1.5 (0.2)		0.38 (0.1)	.02	2.1 (0.2)		0.6 (0.3)	.08

^a^*P* values are based on the unpaired *t* test and by the *χ^2^* test for qualitative variables.

**Table 2:** Comparison of baseline characteristics in the training and test datasets (N=2259).

Variable			Training set (n=1581)		Test set (n=678)		*P* value^a^
**Marital status, n (%)**							.70
	Single	239 (15.1)		95 (14.0)			
	Married	1279 (80.9)		550 (81.1)			
	Divorced	30 (1.9)		17 (2.5)			
	Widowed	33 (2.1)		16 (2.4)			
**Gender, n (%)**							.96
	Men	622 (39.3)		266 (39.2)			
	Women	959 (60.7)		412 (60.8)			
**Cancer history, n (%)**							.38
	No	5 (0.3)		4 (0.6)			
	Yes	1576 (99.7)		674 (99.4)			
**Smoking, n (%)**							.81
	Never	178 (11.3)		72 (10.6)			
	Current/past	1403 (88.7)		606 (89.4)			
**Hospitalization, n (%)**							.59
	No	31 (2.0)		11 (1.6)			
	Yes	1550 (98.0)		667 (98.4)			
**Diabetes, n (%)**							.26
	No	1514 (95.8)		642 (94.7)			
	Yes	67 (4.2)		36 (5.3)			
**Education, n (%)**							.49
	Higher than diploma	95 (6.0)		34 (5.0)			
	Diploma/below diploma	788 (49.8)		330 (48.7)			
	Illiterate/primary school	698 (44.1)		314 (46.3)			
Age (years), mean (SD)			44.4 (11.7)		44.1 (12.2)		.34
BMI (kg/m^2^), mean (SD)			26.8 (4.4)		26.8 (4.4)		.70
Energy (kilocalories), mean (SD)			2278.6 (811.6)		2326.3 (1239.3)		.90
Protein (g), mean (SD)			86.3 (35.7)		87.2 (51.1)		.35
Carbohydrates (g), mean (SD)			338.1 (124.2)		346.3 (215.6)		.81
Monosaturated fatty acids (g), mean (SD)			25.2 (12.5)		25.6 (13.6)		.93
Total fat (g), mean (SD)			74.6 (32.3)		75.9 (37.7)		.92
Carotenoids (mg), mean (SD)			1231.2 (1246.76)		1226.45 (1029.22)		.54
Calcium (mg),mean (SD)			1379.6 (628.8)		1385.5 (681.9)		.65
Magnesium (mg), mean (SD)			471.1 (186.1)		478.0 (367.9)		.30
Zinc (mg), mean (SD)			13.5 (9.6)		13.2 (9.5)		.24
Total fiber (g), mean (SD)			43.5 (20.0)		44.5 (32.9)		.71
Glucose (g), mean (SD)			17.8 (9.5)		18.3 (11.0)		.40
Fructose (g), mean (SD)			21.1 (11.6)		21.6 (13.4)		.52
Sodium (mg), mean (SD)			3464.8 (1578.6)		4699.3 (29481.7)		.34
Folate (mg), mean (SD)			559.9 (202.5)		570.1 (275.3)		.86

^a^*P* values are based on the *t* test for quantitative variables and on the *χ^2^* test for qualitative variables.

### RF Model

The variable importance obtained by the training set using RF is presented in Table 3, showing the results for each variable when all variables were used as input in the RF algorithm. Here, the variable importance was determined by the average decrease in the Gini index. Based on variable importance, the most important determinants of MetS were diabetes, BMI, age, marital status, monounsaturated fatty acids, female gender, and total fat. According to multiple logistic regression analysis, the direction of the association for these variables was as follows: history of diabetes (odd ratio [OR] 6.32, 95% CI 3.92-10.20; *P*<.001), increased BMI (OR 1.19, 95% CI 1.15-1.22; *P*<.001), increased age (OR 1.02, 95% CI 1.01-1.03; *P*<.001), female gender (OR 0.50, 95% CI 0.38-0.63; *P*<.001), and increased dietary monounsaturated fatty acid (OR 0.97, 95% CI 0.94-0.99, *P*=.04) (Multimedia Appendix 4 and Table 3).

History of diabetes (OR=6.32, 95% CI: 3.92, 10.20; *P*<.001), increased BMI (OR=1.19, 95% CI: 1.15, 1.22; *P*<.001), increased age (OR=1.02, 95% CI: 1.01, 1.03; *P*<.001), female gender (OR=0.50, 95% CI: 0.38, 0.63; *P*<.001), and increased monounsaturated fatty acid (OR=0.97, 95% CI: 0.94, 0.99, *P*=.04) (Multimedia Appendix 4 and Table 3).

We obtained an overall out-of-bag correct classification of 98.67% (Table 4). The proportion of error for subjects with and without MetS was 99.24% and 96.55%, respectively.

**Table 3:** Variable importance obtained by random forest for predicting metabolic syndrome.

Variable	Variable importance
Diabetes	100
BMI	67.8
Age	25.2
Gender	15.8
Monosaturated fatty acids	13.9
Carotenoids	13.6
Education	12.5
Calcium	12.0
Protein	10.7
Total Fiber	10.7
Sodium	9.8
Total fat	9.4
Folates	8.9
Zinc	8.8
Magnesium	8.8
Smoking	8.6
Energy	7.9
Carbohydrates	7.8
Fructose	7.6
Hospitalization	7.0
Cancer history	6.9
Marriage	6.9
Glucose	6.6

**Table 4:** Out-of-bag correct classification rates.

Predicted status	Actual status			Correct classification rate
	MetS^b^	No MetS		
MetS	140	5	96.6	
No MetS	4	529	99.3	

^a^MetS: metabolic syndrome.

### Evaluation Criteria

The RF algorithm had high sensitivity (0.97) and specificity (0.99) for the test set. The NPV and PPV performance of RF for the test set were 0.99 and 0.96, respectively. Both the LR+ (103.83) and LR– (0.03) for the test set showed the high ability of the RF algorithm to predict a correct diagnosis of MetS.

Finally, partial plots provided the marginal effect of predictors on MetS (Multimedia Appendix 5).

## Discussion

### Principal Findings

In this prospective study, the total incidence rate of MetS was 229.98 per 1000 person-years. The most important determinants of MetS were a history of diabetes, increased BMI, older age, male gender, and low dietary monounsaturated fatty acid intake.

In this study, diabetes was identified as the most important risk factor (ranking first) for MetS. This finding is expected to be associated with common risk factors of diabetes and MetS (eg, increased BMI, hypertension, high-fat diet, and insulin resistance–linked obesity). In addition, some analytical studies have shown that MetS predicts diabetes independently of other factors [30]. Another study showed that MetS was associated with a 3 to 5-fold increase in the risk of developing type 2 diabetes mellitus [31].

BMI was identified as the second most important risk factor for the incidence of MetS. The development of insulin resistance and the role of inflammatory mediators in MetS are the most important mechanisms in the pathogenesis of obesity. Various studies have shown relationships among hyperinsulinemia, insulin resistance, and increased inflammatory mediators such as C-reactive protein with the development and progression of MetS [14,17,32].

Increased age was the third-ranking factor that was associated with MetS in this study. Aging usually leads to decreased physical activity, followed by an increase in BMI, which can contribute to MetS. Previous studies showed that less than 10% of people in their 20s and 30s were affected by MetS, whereas MetS affected 40% of those over 60 years of age [33,34].

Male gender was the fourth-ranking factor associated with MetS. We observed a significantly higher incidence of MetS among men than among women (27% vs 20%). Although previous studies in Iran showed that the prevalence of MetS was higher among women than among men [35,36], more recent studies confirm our findings, demonstrating the opposite pattern [7]. One reason behind this phenomenon may be the higher prevalence of basic MetS-related characteristics in the men of our study, such as hypertension, higher waist-hip ratio, and higher triglyceride levels.

A low monounsaturated fatty acid intake was identified as the fifth most important factor for a lower occurrence of MetS. Our result is consistent with a recent systematic review that reported that a diet with decreased monounsaturated fats was associated with improving lipoprotein profiles and triglyceride levels [37]. As mentioned earlier, hyperlipidemia is one of the components of MetS. Thus, this finding is consistent with other studies in this area.

### Strengths and Limitations

This study used a population-based cohort (as the gold standard in observational studies) designed based on standard tools for measuring clinical and other variables. This study had some limitations. First, the role of socioeconomic status as an important factor influencing the dietary pattern of subjects was not determined; however, this study was performed on people living in District 13 of Tehran, which is classified as an area with an average income level.

Another limitation of this study was use of the FFQ. Completing a long list of foods consumed over the past year has the potential for recall bias and consequently measurement error, which may distort the results [38,39]. Another important factor for the incidence of MetS is physical activity status; this variable was not included in the analysis due to the large number of missing data.

Finally, the main strength of this study was that the most important risk factors and nutritional factors were ranked. In contrast, previous studies often investigated the predictive factors using classical approaches and neglected the importance of paying attention to risk/protective factors by considering the ranking of the impact of each factor on the outcome. Therefore, lifestyle modification (eg, having a balanced weight and healthy diet) is one of the most important ways to reduce the incidence of MetS.

### Conclusion

In summary, our findings show that the incidence rate of MetS in Tehran was 229.98 per 1000 person-years. The most important determinants of MetS were history of diabetes, increased BMI, increased age, male gender, and decreased dietary monounsaturated fatty acid.

## Appendix

Multimedia Appendix 1 Short summary profile of the Tehran Lipid and Glucose Study (TLGS).

Multimedia Appendix 2 Food Frequency Questionnaire (FFQ) "Tehran Lipid and Glucose Study."

Multimedia Appendix 3 Formulas used in this study for model evaluation.

Multimedia Appendix 4 Influence of nutritional and other predictors for developing MetS in the whole population based on the multivariable logistic regression model.

Multimedia Appendix 5 The partial plots of variables that presented variable importance.

## Abbreviations

FFQ: Food Frequency Questionnaire
HLDL-C: high-density lipoprotein cholesterol
LR: likelihood ratio
MetS: metabolic syndrome
NPV: negative predictive value
OR: odds ratio
PPV: positive predictive value
RF: random forest

## References

[1] Cardiology Research and Practice (2019). Retracted: A comprehensive review on metabolic syndrome. Cardiol Res Pract.

[2] Alberti KGMM, Zimmet P, Shaw J (2006). Metabolic syndrome--a new world-wide definition. A Consensus Statement from the International Diabetes Federation. Diabet Med.

[3] Ford ES, Giles WH, Dietz WH (2002). Prevalence of the metabolic syndrome among US adults: findings from the third National Health and Nutrition Examination Survey. JAMA.

[4] Ranasinghe P, Mathangasinghe Y, Jayawardena R, Hills AP, Misra A (2017). Prevalence and trends of metabolic syndrome among adults in the Asia-Pacific region: a systematic review. BMC Public Health.

[5] Kolovou GD, Anagnostopoulou KK, Salpea KD, Mikhailidis DP (2007). The prevalence of metabolic syndrome in various populations. Am J Med Sci.

[6] Fatahi A, Doosti-Irani A, Cheraghi Z (2020). Prevalence and incidence of metabolic syndrome in Iran: a systematic review and meta-analysis. Int J Prev Med.

[7] Hadaegh F, Hasheminia M, Lotfaliany M, Mohebi R, Azizi F, Tohidi M (2013). Incidence of metabolic syndrome over 9 years follow-up; the importance of sex differences in the role of insulin resistance and other risk factors. PLoS One.

[8] Ghotboddin Mohammadi S, Mirmiran P, Bahadoran Z, Mehrabi Y, Azizi F (2015). The association of dairy intake with metabolic syndrome and its components in adolescents: Tehran Lipid and Glucose Study. Int J Endocrinol Metab.

[9] Cheraghi Z, Nedjat S, Mirmiran P, Moslehi N, Mansournia N, Etminan M, Mansournia MA, McCandless LC (2018). Effects of food items and related nutrients on metabolic syndrome using Bayesian multilevel modelling using the Tehran Lipid and Glucose Study (TLGS): a cohort study. BMJ Open.

[10] Cheraghi Z, Mirmiran P, Mansournia MA, Moslehi N, Khalili D, Nedjat S (2016). The association between nutritional exposures and metabolic syndrome in the Tehran Lipid and Glucose Study (TLGS): a cohort study. Public Health.

[11] Bagry HS, Raghavendran S, Carli F (2008). Metabolic syndrome and insulin resistance: perioperative considerations. Anesthesiology.

[12] Kohavi R, Quinlan J, Klösgen W, Zytkow JM (2002). Data mining tasks and methods: Classification: decision-tree discovery. Handbook of data mining and knowledge discovery.

[13] Krishnaiah V, Narsimha G, Chandra D (2013). Diagnosis of lung cancer prediction system using data mining classification techniques. Int J Comput Sci Inf Technol.

[14] Yu C, Lin Y, Lin C, Wang S, Lin S, Lin SH, Wu JL, Chang S (2020). Predicting metabolic syndrome with machine learning models using a decision tree algorithm: retrospective cohort study. JMIR Med Inform.

[15] Azizi F, Rahmani M, Emami H, Mirmiran P, Hajipour R, Madjid M, Ghanbili J, Ghanbarian A, Mehrabi Y, Saadat N, Salehi P, Mortazavi N, Heydarian P, Sarbazi N, Allahverdian S, Saadati N, Ainy E, Moeini S (2002). Cardiovascular risk factors in an Iranian urban population: Tehran lipid and glucose study (phase 1). Soz Praventivmed.

[16] Hosseini-Esfahani F, Bahadoran Z, Moslehi N, Asghari G, Yuzbashian E, Hosseinpour-Niazi S, Mirmiran P, Azizi F (2018). Metabolic syndrome: findings from 20 years of the Tehran Lipid and Glucose Study. Int J Endocrinol Metab.

[17] Alberti KGMM, Eckel RH, Grundy SM, Zimmet PZ, Cleeman JI, Donato KA, Fruchart J, James WPT, Loria CM, Smith SC, International Diabetes Federation Task Force on EpidemiologyPrevention, Hational Heart‚ Lung‚Blood Institute, American Heart Association, World Heart Federation, International Atherosclerosis Society, International Association for the Study of Obesity (2009). Harmonizing the metabolic syndrome: a joint interim statement of the International Diabetes Federation Task Force on Epidemiology and Prevention; National Heart, Lung, and Blood Institute; American Heart Association; World Heart Federation; International Atherosclerosis Society; and International Association for the Study of Obesity. Circulation.

[18] Azizi F, Hadaegh F, Khalili D, Esteghamati A, Hosseinpanah F, Delavari A, Larijani B, Mirmiran P, Zabetian A, Mehrabi Y, Kelishadi R, Aghajani H (2010). Appropriate definition of metabolic syndrome among Iranian adults: report of the Iranian National Committee of Obesity. Arch Iran Med.

[19] Malekshah AF, Kimiagar M, Saadatian-Elahi M, Pourshams A, Nouraie M, Goglani G, Hoshiarrad A, Sadatsafavi M, Golestan B, Yoonesi A, Rakhshani N, Fahimi S, Nasrollahzadeh D, Salahi R, Ghafarpour A, Semnani S, Steghens JP, Abnet CC, Kamangar F, Dawsey SM, Brennan P, Boffetta P, Malekzadeh R (2006). Validity and reliability of a new food frequency questionnaire compared to 24 h recalls and biochemical measurements: pilot phase of Golestan cohort study of esophageal cancer. Eur J Clin Nutr.

[20] Mirmiran P, Hosseini Esfahani F, Mehrabi Y, Hedayati M, Azizi F (2009). Reliability and relative validity of an FFQ for nutrients in the Tehran Lipid and Glucose Study. Public Health Nutr.

[21] Esfahani FH, Asghari G, Mirmiran P, Azizi F (2010). Reproducibility and relative validity of food group intake in a food frequency questionnaire developed for the Tehran Lipid and Glucose Study. J Epidemiol.

[22] Ford ES, Ajani UA, McGuire LC, Liu S (2005). Concentrations of serum vitamin D and the metabolic syndrome among U.S. adults. Diabetes Care.

[23] Ford ES, Mokdad AH, Giles WH, Brown DW (2003). The metabolic syndrome and antioxidant concentrations: findings from the Third National Health and Nutrition Examination Survey. Diabetes.

[24] Esmaillzadeh A, Kimiagar M, Mehrabi Y, Azadbakht L, Hu FB, Willett WC (2006). Fruit and vegetable intakes, C-reactive protein, and the metabolic syndrome. Am J Clin Nutr.

[25] Breiman L (2001). Random forests. Machine Lang.

[26] Breiman L (2001). Statistical modeling: the two cultures (with comments and a rejoinder by the author). Statist Sci.

[27] Meyer D, Leisch F, Hornik K (2003). The support vector machine under test. Neurocomputing.

[28] Verikas A, Gelzinis A, Bacauskiene M (2011). Mining data with random forests: A survey and results of new tests. Pattern Recognition.

[29] Aličković E, Subasi A (2015). Breast cancer diagnosis using GA feature selection and rotation forest. Neural Comput Applic.

[30] Lorenzo C, Okoloise M, Williams K, Stern MP, Haffner SM, San Antonio Heart Study (2003). The metabolic syndrome as predictor of type 2 diabetes: the San Antonio heart study. Diabetes Care.

[31] Wilson PWF, D'Agostino RB, Parise H, Sullivan L, Meigs JB (2005). Metabolic syndrome as a precursor of cardiovascular disease and type 2 diabetes mellitus. Circulation.

[32] Wang H, Steffen LM, Vessby B, Basu S, Steinberger J, Moran A, Jacobs DR, Hong C, Sinaiko AR (2011). Obesity modifies the relations between serum markers of dairy fats and inflammation and oxidative stress among adolescents. Obesity (Silver Spring).

[33] Morley J (2004). The metabolic syndrome and aging. J Gerontol A Biol Sci Med Sci.

[34] Bonomini F, Rodella LF, Rezzani R (2015). Metabolic syndrome, aging and involvement of oxidative stress. Aging Dis.

[35] Zabetian A, Hadaegh F, Azizi F (2007). Prevalence of metabolic syndrome in Iranian adult population, concordance between the IDF with the ATPIII and the WHO definitions. Diabetes Res Clin Pract.

[36] Janghorbani M, Amini M (2012). Incidence of metabolic syndrome and its risk factors among type 2 diabetes clinic attenders in Isfahan, Iran. ISRN Endocrinol.

[37] Garg A (1998). High-monounsaturated-fat diets for patients with diabetes mellitus: a meta-analysis. Am J Clin Nutr.

[38] Kristal AR, Peters U, Potter JD (2005). Is it time to abandon the food frequency questionnaire?. Cancer Epidemiol Biomarkers Prev.

[39] Lutsey PL, Steffen LM, Stevens J (2008). Dietary intake and the development of the metabolic syndrome: the Atherosclerosis Risk in Communities study. Circulation.

