# Supplementary material for: Using Machine Learning Techniques to Predict Factors Contributing to the Incidence of Metabolic Syndrome in Tehran: Cohort Study
Source: JMIR Public Health Surveill. 2021 Sep 2;7(9):e27304. doi: 10.2196/27304 (PMC8446845; doi:10.2196/27304)
Supplement: Multimedia Appendix 1 [file publichealth_v7i9e27304_app1.docx]

The Tehran Lipid and Glucose Study is a study to determine the risk factors for atherosclerosis among the Tehran urban population and develop population-based measures to change the lifestyle of the population and reduce the incidence of diabetes mellitus and dyslipidemia. A total of 17,000 men and women between 3 and 69 years of age are randomly sampled from Tehran urban district 13 for assessment of conventional (i.e. smoking, diabetes and hypertension) and emerging (i.e. glucose intolerance, obesity, physical activity and diet) cardiovascular risk factors. In the baseline examination, a general questionnaire on health condition and risk factors is completed and a brief physical examination is done. Cardiovascular disease is assessed using a 12-lead electrocardiogram and London School of Hygiene Cardiovascular (Rose) Questionnaire. The relationship between the conventional and emerging risk factors, vascular disease, and markers of end-organ damage will be evaluated in the urban residents of Tehran. In the second phase of the study, healthy individuals will be followed up in a 10-year-long cohort to determine the trend of changes in lipid and glucose status and development of vascular complications. Selected participants will undergo interventions with modifications in their lifestyle to assess the efficacy of certain measures in preventing the development of diabetes mellitus and dyslipidemia.
