# Supplementary material for: Using Machine Learning Techniques to Predict Factors Contributing to the Incidence of Metabolic Syndrome in Tehran: Cohort Study
Source: JMIR Public Health Surveill. 2021 Sep 2;7(9):e27304. doi: 10.2196/27304 (PMC8446845; doi:10.2196/27304)
Supplement: Multimedia Appendix 2 [file publichealth_v7i9e27304_app2.docx]

Appendix 2: Food Frequency Questionnaire (FFQ) "Tehran Lipid and Glucose Study"

Acceptance Code: Age:

| No. | DAIRY FOODS | HOW MUCH |  | HOW | OFTEN |  | CONSIDERATIONS |
| --- | --- | --- | --- | --- | --- | --- | --- |
|  |  |  | per day | per week | per month | per year |  |
| 1 | Lavash bread (refined grains) | 1 loaf |  |  |  |  |  |
| 2 | Barbari bread (refined grains) | 1 loaf |  |  |  |  |  |
| 3 | Sangak bread (whole grains) | 1 loaf |  |  |  |  |  |
| 4 | Taftoon bread (refined grains) | 1 loaf |  |  |  |  |  |
| 5 | Baguette bread | 1 loaf |  |  |  |  |  |
| 6 | Cooked rice | 1 dinner plate |  |  |  |  | normal: full: |
| 7 | Cooked pasta | 1 spatula |  |  |  |  | Flat head: full: |
| 8 | Potato | 1 medium |  |  |  |  |  |
| 9 | French fries | 1 slice |  |  |  |  |  |
| 10 | Baked vermicelli (soup noodle) | 1 cup |  |  |  |  |  |
| 11 | Ash noodle | 1 cup |  |  |  |  |  |
| 12 | Cookies (stating the type) | 1 number |  |  |  |  | type: |
| 13 | Crackers (crispy biscuits) | 1 number |  |  |  |  |  |
| 14 | Types of cakes | 1 medium slice |  |  |  |  | (Including homemade cakes, cookies, T-tops, etc.) |
| 15 | Corn | 1 medium |  |  |  |  |  |
| 16 | Cooked barley or bulgur | 1 cup |  |  |  |  |  |
| 17 | Lentil | 1 cup |  |  |  |  |  |
| 18 | Beans | 1 cup |  |  |  |  |  |
| 19 | Pea | 1 cup |  |  |  |  |  |
| 20 | Baked bean | 1 cup |  |  |  |  |  |
| 21 | Soy bean | 1 cup |  |  |  |  |  |
| 22 | Mung | 1 cup |  |  |  |  |  |
| 23 | Cotyledon | 1 cup |  |  |  |  |  |
| 24 | Beef or calf | 1 slice of stewed |  |  |  |  |  |
| 25 | lamb meat | 1 slice of stewed |  |  |  |  |  |
| 26 | Ground beef | 1 tablespoon |  |  |  |  |  |
| 27 | Hen and Chickens | 1 medium piece |  |  |  |  | Drumstick: chest: wings: with peel: peeled: |
| 28 | Fish (except tuna) stating the type | 1 medium piece (one palm full) |  |  |  |  | The amount of: type: |
| 29 | Tuna (canned) | 1/2 cans |  |  |  |  | Is it discarded oil? Yes * No * |
| 30 | Heart, liver and kidney | 1 skewer |  |  |  |  |  |
| 31 | Hamburger | 1 number |  |  |  |  |  |
| 32 | kielbasa | 1 Cut |  |  |  |  |  |
| 33 | Sausage | 1 number (Germany * cocktails *) |  |  |  |  |  |
| 34 | Egg | 1 Number |  |  |  |  |  |
| 35 | Tripe and Rennet | 1 piece |  |  |  |  |  |
| 36 | Tongue | 1 whole number |  |  |  |  |  |
| 37 | Brain | 1 whole number |  |  |  |  |  |
| 38 | Head | 1 palm |  |  |  |  |  |
| 39 | Leg | 1 Number |  |  |  |  | If certain amount and components is used, noted |
| 40 | Pizza | 1 number |  |  |  |  |  |
| 41 | Low-fat milk (less than 2%) | 1 cup |  |  |  |  |  |
| 42 | Whole milk (greater or equal to 2%) | 1 cup |  |  |  |  |  |
| 43 | Chocolate milk | 1 cup |  |  |  |  |  |
| 44 | Ooze Yogurt | 1 tablespoon |  |  |  |  |  |
| 45 | Regular yogurt | 1 Bowl/cup |  |  |  |  |  |
| 46 | Full fat yogurt | 1 Bowl/cup |  |  |  |  |  |
| 47 | Cheese | 1 pat |  |  |  |  |  |
| 48 | Cream cheese | 1 pat |  |  |  |  |  |
| 49 | Dough | 1 cup |  |  |  |  |  |
| 50 | Cream | 1 tablespoon |  |  |  |  |  |
| 51 | Traditional ice cream | Half cup |  |  |  |  | how months of year? |
| 52 | Non-traditional ice cream | 1 number |  |  |  |  | how months of year? |
| 53 | Butter | 1 pat |  |  |  |  |  |
| 54 | Margarine | 1 pat |  |  |  |  |  |
| 55 | Dried whey | 1 tablespoon |  |  |  |  |  |
| 56 | Shredded lettuce | 1 cup |  |  |  |  |  |
| 57 | Tomato | 1 medium |  |  |  |  |  |
| 58 | Cucumber | 1 medium |  |  |  |  |  |
| 59 | Fresh Herbs | 1 small plate |  |  |  |  |  |
| 60 | Cooked vegetables (soup, rice, etc.) | 1 cup |  |  |  |  |  |
| 61 | Pumpkin | 1 medium |  |  |  |  |  |
| 62 | Stewed pumpkin | 1 medium |  |  |  |  |  |
| 63 | Baked Eggplant | 1 medium |  |  |  |  |  |
| 64 | Boiled Celery | 1 cup |  |  |  |  |  |
| 65 | Green peas cooked | 1 cup |  |  |  |  |  |
| 66 | Green beans cooked | 1 cup |  |  |  |  |  |
| 67 | Raw carrots | 1 medium |  |  |  |  |  |
| 68 | Cooked carrots | 1 medium |  |  |  |  |  |
| 69 | Garlic | 1 clove |  |  |  |  |  |
| 70 | Raw onion | 1 small |  |  |  |  |  |
| 71 | Fried onions | 1 tablespoon |  |  |  |  |  |
| 72 | Cabbage varieties | 1 Bowl/cup |  |  |  |  |  |
| 73 | Bell peppers | 1 medium |  |  |  |  |  |
| 74 | Cooked spinach | 1 cup |  |  |  |  |  |
| 75 | Turnip | 1 medium |  |  |  |  |  |
| 76 | Small green pepper | 1 medium |  |  |  |  |  |
| 77 | ketchup | 1 tablespoon |  |  |  |  |  |
| 78 | Pickles | 1 cup |  |  |  |  | how months of year? |
| 79 | SHOOR | 1 cup |  |  |  |  | how months of year? |
| 80 | Pickled cucumber | 1 medium |  |  |  |  |  |
| 81 | Cantaloupe | 1/4 number |  |  |  |  |  |
| 82 | Melon | 1 medium slice |  |  |  |  |  |
| 83 | Watermelon | 1 medium slice |  |  |  |  |  |
| 84 | Pear | 1 medium |  |  |  |  |  |
| 85 | Apricot | 1 medium |  |  |  |  |  |
| 86 | Cherries | 1 small plate |  |  |  |  |  |
| 87 | Apple | 1 medium |  |  |  |  |  |
| 88 | Peach | 1 medium |  |  |  |  |  |
| 89 | Nectarines | 1 medium |  |  |  |  |  |
| 90 | Prunus | 1 medium |  |  |  |  |  |
| 91 | Fresh figs | 1 medium |  |  |  |  |  |
| 92 | Dried figs | 1 medium |  |  |  |  |  |
| 93 | Grape | 1 medium bunch |  |  |  |  |  |
| 94 | Kiwi | 1 medium |  |  |  |  |  |
| 95 | Grapefruit | 1 medium |  |  |  |  |  |
| 96 | Orange | 1 medium |  |  |  |  |  |
| 97 | Persimmon | 1 medium |  |  |  |  |  |
| 98 | Tangerine | 1 medium |  |  |  |  |  |
| 99 | Pomegranate | 1 medium |  |  |  |  |  |
| 100 | Date | 1 medium |  |  |  |  |  |
| 101 | Plums (yellow and red) | 1 medium |  |  |  |  |  |
| 102 | Strawberry | 1 number |  |  |  |  |  |
| 103 | Banana | 1 medium |  |  |  |  |  |
| 104 | Sweet lemon | 1 medium |  |  |  |  |  |
| 105 | Lemon | 1 medium |  |  |  |  |  |
| 106 | Orange juice | 1 cup |  |  |  |  |  |
| 107 | Apple juice | 1 cup |  |  |  |  |  |
| 108 | Cantaloupe juice | 1 cup |  |  |  |  |  |
| 109 | Raisins | 1 tablespoon |  |  |  |  |  |
| 110 | Fresh berries | 1 small plate |  |  |  |  |  |
| 111 | Dried berries | 1 number |  |  |  |  |  |
| 112 | Dried fruits | 1 number |  |  |  |  |  |
| 113 | Green Olive | 1 number |  |  |  |  |  |
| 114 | Canned fruits | 1 can |  |  |  |  |  |
| 115 | Packaged fruit juices | 1 number |  |  |  |  |  |
| 116 | Solid vegetables oils | 1 tablespoon |  |  |  |  |  |
| 117 | Oil | 1 tablespoon |  |  |  |  |  |
| 118 | Olive oil | 1 tablespoon |  |  |  |  |  |
| 119 | Ghee | 1 tablespoon |  |  |  |  |  |
| 120 | Mayonnaise | 1 tablespoon |  |  |  |  |  |
| 121 | Peanut | 1 number |  |  |  |  |  |
| 122 | Almond | 1 number |  |  |  |  |  |
| 123 | Walnut | 1whole number |  |  |  |  |  |
| 124 | Pistachios | 1 number |  |  |  |  |  |
| 125 | Hazelnut | 1 number |  |  |  |  |  |
| 126 | Seeds (watermelon, pumpkin, sunflower) | 1 Bowl/cup |  |  |  |  |  |
| 127 | Sugar cube, comfit | 1 number |  |  |  |  |  |
| 128 | Sugar | 1 teaspoonful |  |  |  |  |  |
| 129 | Honey | 1 teaspoonful |  |  |  |  |  |
| 130 | Jams (by type) | 1 tablespoon |  |  |  |  |  |
| 131 | Industrial Or cola beverages. | 1 cup |  |  |  |  |  |
| 132 | Dried sweets | 1 medium |  |  |  |  |  |
| 133 | Cream Sweets | 1 medium |  |  |  |  |  |
| 134 | GAZ | 1 medium |  |  |  |  |  |
| 135 | SOHAN | 1 Piece |  |  |  |  |  |
| 136 | Puff | 1 pack |  |  |  |  |  |
| 137 | Chocolate | 1 number |  |  |  |  |  |
| 138 | Tea | 1 cup |  |  |  |  |  |
| 139 | Salt | 1 tsp |  |  |  |  | Type: |
| 140 | Chips | 1 pack |  |  |  |  |  |
| 141 | Coffee | 1 cup |  |  |  |  |  |
| 142 | Lemon juice | 1 teaspoonful |  |  |  |  |  |
| 143 | Candy | 1 medium |  |  |  |  |  |
| 144 | Baked mushrooms | Half cup |  |  |  |  |  |
| 145 | Homemade HALVA | 1 tablespoon |  |  |  |  |  |
| 146 | Sesame pudding | 1 tablespoon |  |  |  |  |  |
| 147 | Spices | 1 tsp |  |  |  |  |  |

The date of delivery of food questionnaires to residents:

Name of dietitian responsible for completing FFQ:

delivery date to Nutrition expert:
