# Supplementary material for: Using Machine Learning Techniques to Predict Factors Contributing to the Incidence of Metabolic Syndrome in Tehran: Cohort Study
Source: JMIR Public Health Surveill. 2021 Sep 2;7(9):e27304. doi: 10.2196/27304 (PMC8446845; doi:10.2196/27304)
Supplement: Multimedia Appendix 3 [file publichealth_v7i9e27304_app3.docx]

$$Sensitivity=\frac{TP}{TP+FN}$$

$$\mathrm{specificity}=\frac{TN}{TN+FP}$$

$$NPV=\frac{TN}{TN+FN}$$

$$PPV=\frac{TP}{TP+FP}$$

$$LR+=\frac{SEN}{1-SPE}$$

$$LR-=\frac{1-SEN}{SPE}$$

Where TP, FP, TN, and FN are true positive, false positive, true negative, and false negative, respectively. Sensitivity is the ability of a test to correctly recognize the subjects with MetS, while specificity is the ability of a test to recognize the subjects that do not have MetS. A larger value of the positive likelihood ratio (LR+) as well as a smaller value of the negative likelihood ratio (LR–) indicates a greater ability of RF to identify valuable information in a test.
