# Supplementary material for: Using Machine Learning Techniques to Predict Factors Contributing to the Incidence of Metabolic Syndrome in Tehran: Cohort Study
Source: JMIR Public Health Surveill. 2021 Sep 2;7(9):e27304. doi: 10.2196/27304 (PMC8446845; doi:10.2196/27304)
Supplement: Multimedia Appendix 4 [file publichealth_v7i9e27304_app4.docx]

**Influence of nutritional and other predictors for developing MetS in the whole population based on the multivariable logistic regression model**

| **Variables** | **Adjusted** | **p-value** | **95% CI** |
| --- | --- | --- | --- |
|  | **OR** |  |  |
| Age (per year) | 1.02 | **<.001** | [1.01, 1.03] |
| Marriage status (married vs. single) | 1.27 | .25 | [0.84, 1.90] |
| Sex (female vs. male) | 0.5 | **<.001** | [0.38, 0.63] |
| Education (per level) | 0.89 | .15 | [0.76, 1.04] |
| Smoking (yes vs. no) | 1.3 | .13 | [93, 1.83] |
| BMI (per one unit: kg/m2) | 1.19 | **<.001** | [1.15, 1.22] |
| Diabetes (yes vs. no) | 6.32 | **<.001** | [3.92, 10.20] |
| Total Fat (per gr) | 0.98 | .03 | [0.96, 0.99] |
| Monounsaturated-Fatty Acids (per gr) | 0.97 | **.04** | [0.94, 0.99] |
| Energy (kcal) | 1.01 | **.001** | [1.00, 1.02] |
| Protein (per gr) | 0.99 | .13 | [0.98, 1.00] |
| Carbohydrates (per gr) | 0.99 | **.002** | [0.98, 0.99] |
| Sodium (per gr) | 1 | .59 | [0.99, 1.01] |
| Calcium (per gr) | 1 | .05 | [0.99, 1.01] |
| Folates (per gr) | 1 | .08 | [0.99, 1.01] |
| Magnesium (per gr) | 1 | .70 | [0.99, 1.01] |
| Zinc (per gr) | 1.02 | .06 | [0.99, 1.05] |
| Total Fiber (per gr) | 1.02 | **.001** | [1.01, 1.03] |
| Carotenoids (per gr) | 1.01 | **.03** | [1.001, 1.02] |
| Fructose (per gr) | 1.02 | .54 | [0.99, 1.02] |
