# Supplementary figures and images for: Using Machine Learning Techniques to Predict Factors Contributing to the Incidence of Metabolic Syndrome in Tehran: Cohort Study
Source: JMIR Public Health Surveill. 2021 Sep 2;7(9):e27304. doi: 10.2196/27304 (PMC8446845; doi:10.2196/27304)

**Appendix 5: The partial plots of variables that presented in variable importance.**

| *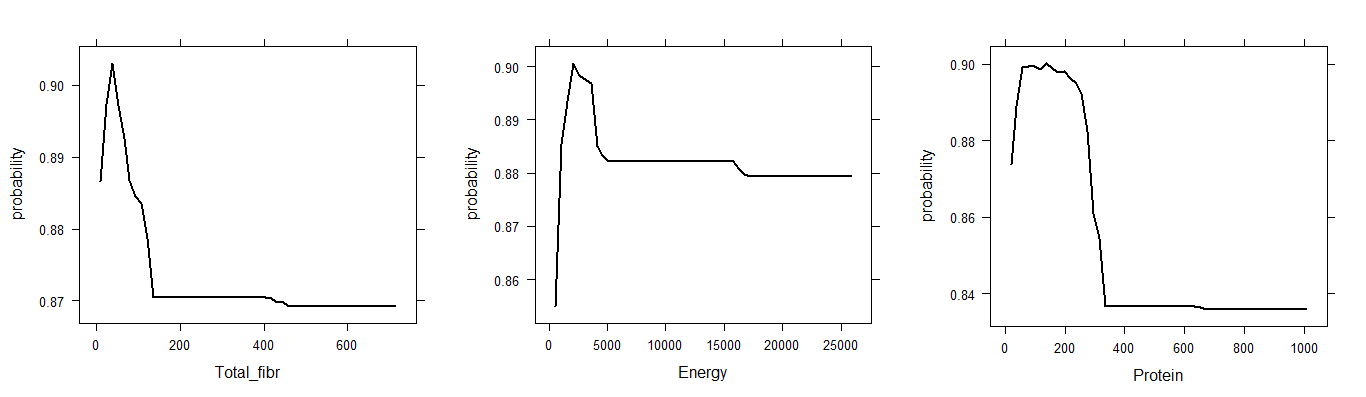* |
| --- |
| *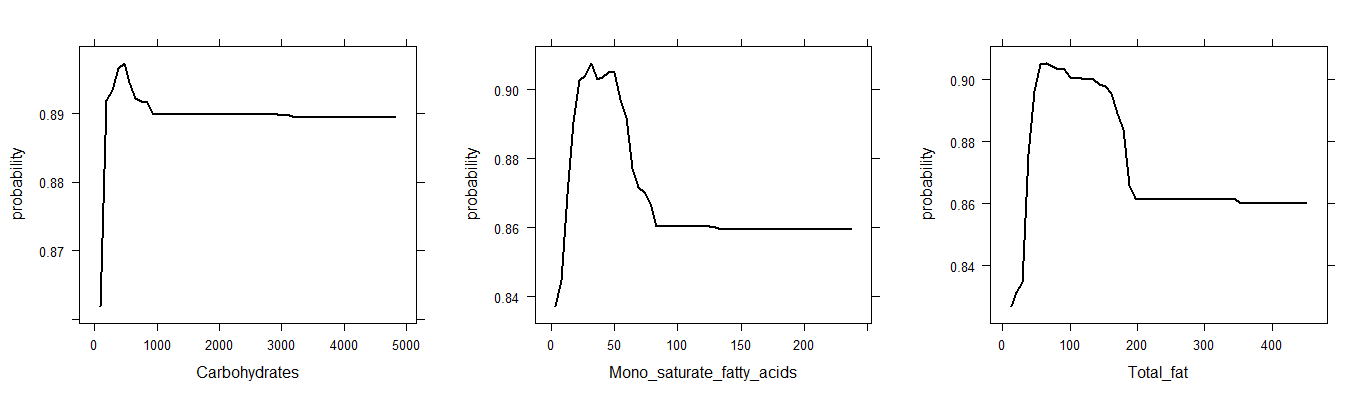* |
| *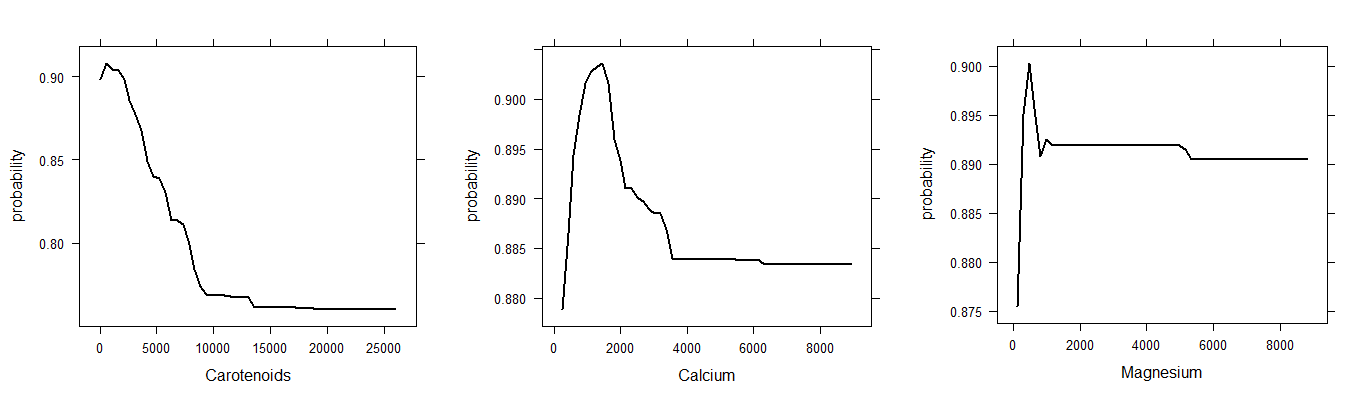* |
| *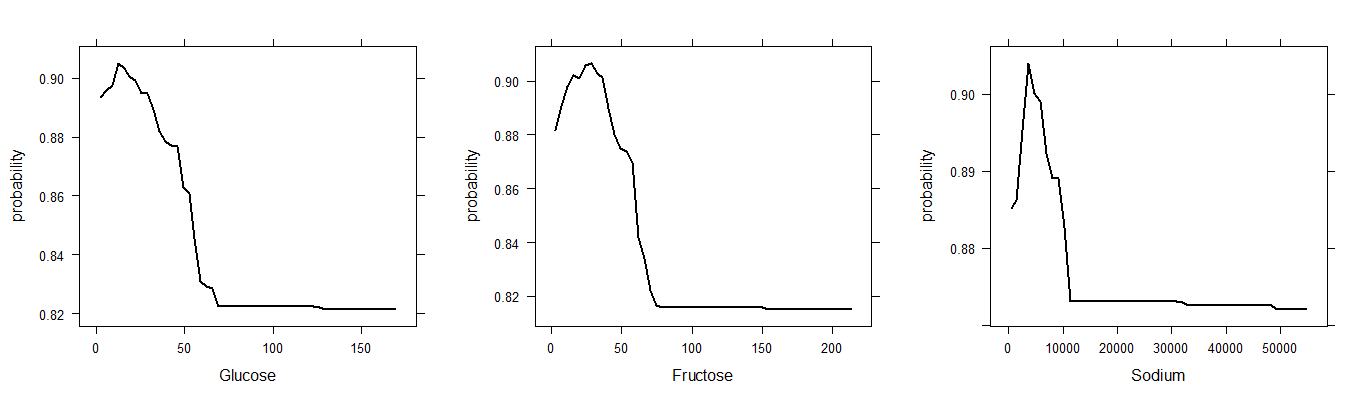* |
| *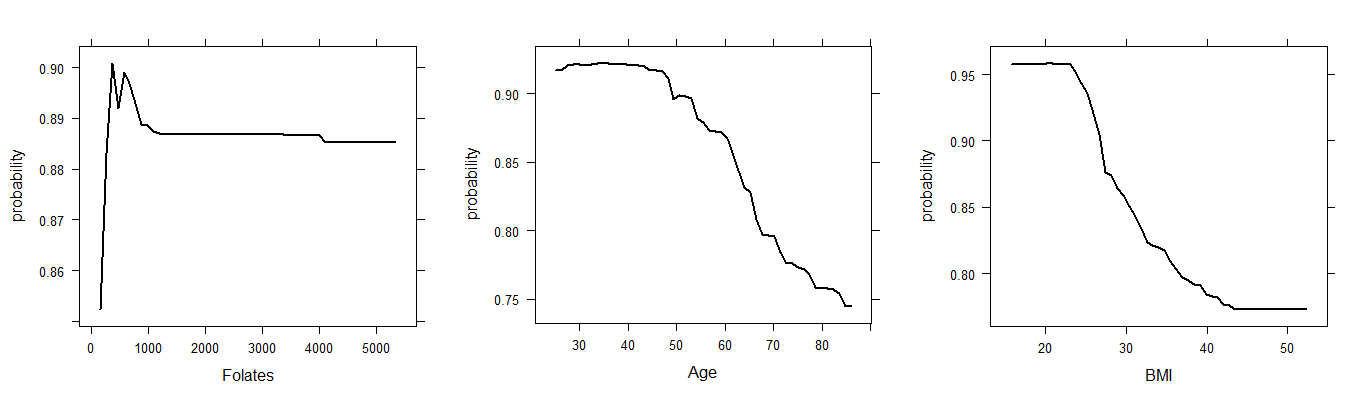* |
| *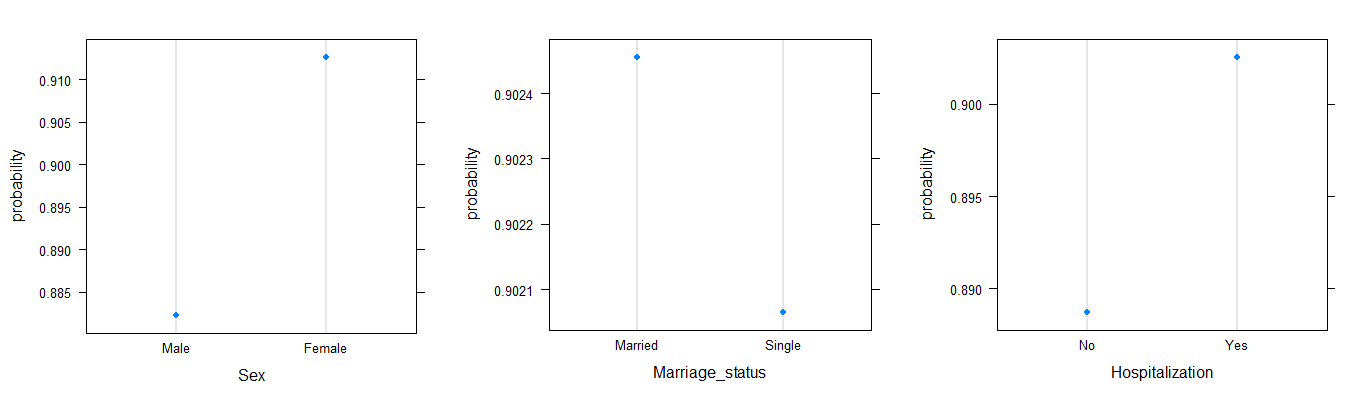* |
| *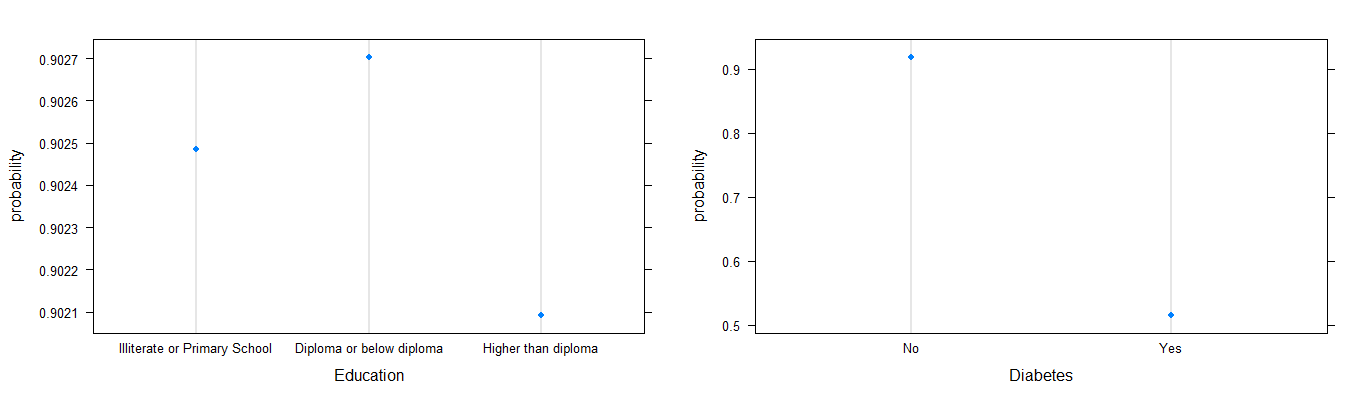* |

Supplement: Multimedia Appendix 5 [file publichealth_v7i9e27304_app5.docx]
